# Tick salivary gland extract induces alpha‐gal syndrome in alpha‐gal deficient mice

**DOI:** 10.1002/iid3.457

**Published:** 2021-05-25

**Authors:** Shailesh K. Choudhary, Shahid Karim, Onyinye I. Iweala, Shivangi Choudhary, Gary Crispell, Surendra Raj Sharma, Claire T. Addison, Mike Kulis, Brian H. Herrin, Susan E. Little, Scott P. Commins

**Affiliations:** ^1^ Division of Allergy, Immunology and Rheumatology, Thurston Arthritis Research Center University of North Carolina Chapel Hill North Carolina USA; ^2^ Center for Molecular and Cellular Biosciences, Department of Cell and Molecular Biology, School of Biological, Environmental, and Earth Sciences The University of Southern Mississippi Hattiesburg Mississippi USA; ^3^ UNC Food Allergy Initiative, Department of Pediatrics University of North Carolina Chapel Hill North Carolina USA; ^4^ Department of Diagnostic Medicine and Pathobiology Kansas State University Manhattan Kansas USA; ^5^ Department of Veterinary Pathobiology Oklahoma State University Stillwater Oklahoma USA

**Keywords:** alpha‐gal, alpha‐gal knockout mice, alpha‐gal syndrome, *Amblyomma americanum*, delayed allergic responses, food allergy, mammalian meat, tick

## Abstract

**Introduction:**

Alpha‐gal syndrome (AGS) is characterized by delayed hypersensitivity to non‐primate mammalian meat in people having specific immunoglobulin E (sIgE) to the oligosaccharide galactose‐alpha‐1,3‐galactose. AGS has been linked to tick bites from *Amblyomma americanum* (*Aa*) in the U.S. A small animal model of meat allergy is needed to study the mechanism of alpha‐gal sensitization, the effector phase leading to delayed allergic responses and potential therapeutics to treat AGS.

**Methods:**

Eight‐ to ten‐weeks old mice with a targeted inactivation of alpha‐1,3‐galactosyltransferase (AGKO) were injected intradermally with 50 μg of *Aa* tick salivary gland extract (TSGE) on days 0, 7, 21, 28, 42, and 49. Total IgE and alpha‐gal sIgE were quantitated on Day 56 by enzyme‐linked immunosorbent assay. Mice were challenged orally with 400 mg of cooked pork kidney homogenate or pork fat. Reaction severity was assessed by measuring a drop in core body temperature and scoring allergic signs.

**Results:**

Compared to control animals, mice treated with TSGE had 190‐fold higher total IgE on Day 56 (0.60 ± 0.12 ng/ml vs. 113.2 ± 24.77 ng/ml; *p* < 0.001). Alpha‐gal sIgE was also produced in AGKO mice following TSGE sensitization (undetected vs. 158.4 ± 72.43 pg/ml). Further, sensitized mice displayed moderate clinical allergic signs along with a drop in core body temperature of ≥2°C as an objective measure of a systemic allergic reaction. Interestingly, female mice had higher total IgE responses to TSGE treatment but male mice had larger declines in mean body temperature.

**Conclusion:**

TSGE‐sensitized AGKO mice generate sIgE to alpha‐gal and demonstrate characteristic allergic responses to pork fat and pork kidney. In keeping with the AGS responses documented in humans, mice reacted more rapidly to organ meat than to high fat pork challenge. This mouse model establishes the central role of tick bites in the development of AGS and provides a small animal model to mechanistically study mammalian meat allergy.

## Graphical abstract

Alpha‐gal syndrome (AGS) is characterized by delayed hypersensitivity to non‐primate mammalian meat in people having specific IgE (sIgE) to the oligosaccharide galactose‐alpha‐1,3‐galactose and has been linked to tick bites from Amblyomma americanum (Aa) in the U.S. We demonstrate that intradermal injection of Aa tick salivary gland extract (TSGE) in alpha‐gal knockout (AGKO) mice induce alpha‐gal sIgE production and mice displayed moderate clinical allergic signs along with a drop in core body temperature as an objective measure of a systemic allergic reaction. Further, this model recapitulates several aspects of red meat allergy seen in the humans and will be used to mechanistically study this novel food allergy and model therapeutic approaches to treat this disease.

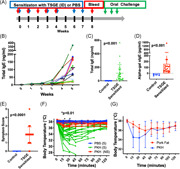

## 1 INTRODUCTION

Alpha‐gal syndrome (AGS) is a unique allergy to the oligosaccharide galactose‐α‐1,3‐galactose (alpha‐gal), which is present in beef, pork, lamb and meat from all other mammals except catarrhine primates (apes and humans).^1^, ^2^ In humans the alpha‐gal moiety is absent because the α1,3GT gene became inactivated in an Old World ancestor.^2^ Nevertheless, the alpha‐gal moiety is of major clinical significance as humans produce a natural antibody (anti‐Gal) as immunoglobulin M, immunoglobulin A, and immunoglobulin G isotypes to this epitope.^2^ AGS, in contrast, is due to an alpha‐gal‐directed specific immunoglobulin E (sIgE) antibody and allergic reactions typically occur 2–6 h after ingestion of “red meat” or derived‐products.^1^, ^3^, ^4^ Although AGS is characterized by a delayed onset allergic reaction, consumption of mammalian organ meat has been associated with a shorter delay before symptoms (<2 h) as well as more consistent reactions.^5^ Since our initial finding, there has been a sharp increase in the number of patients with AGS and it has become the most prominent new‐onset food allergy of adults in the Southeastern U.S.^6^ In fact, a recent analysis of patients presenting with anaphylaxis to a practice in Tennessee found that AGS was the most common etiology, accounting for 33% of cases with all other food allergy diagnoses at 28%.^7^ In keeping with this, Viracor Eurofins (the only national reference lab performing alpha‐gal sIgE testing) recently reported gretaer than 34,000 positive results since 2010.^8^

Interestingly, an alpha‐gal sIgE response can develop after years of safely tolerating mammalian meat and has been linked to the bites of the tick *Amblyomma americanum* (*Aa*, the lone star tick) in the U.S and bites of other species of ticks, such as *Ixodes holocyclus, Ixodes ricinus, Haemaphysalis longicornis*, and *Amblyomma sculptum* in Australia, Europe, Japan, and Brazil, respectively.^9^, ^10^, ^11^, ^12^, ^13^, ^14^ In addition to an epidemiologic correlation between the distribution of *Aa* ticks and the geographic areas where alpha‐gal sIgE antibody has been reported, limited prospective data show a rise in IgE antibody to alpha‐gal following tick bites.^9^ The mechanisms by which *Aa* bites induce alpha‐gal sIgE production and the delayed response to red meat during allergic reactions are poorly understood, owing largely to the absence of a relevant small animal model that truly reflects AGS as observed in humans.

In this study article, we report that mice with a targeted inactivation of the alpha(1,3)‐galactosyltransferase gene (AGKO), which mimic humans as “alpha‐gal‐deficient,” develop alpha‐gal sIgE following intradermal injection with *Aa* tick salivary gland extract (TSGE). This alpha‐gal sIgE response does not require supplementation with an adjuvant or an alpha‐gal‐containing glycoprotein and the mice display an allergic phenotype upon food challenge.

## 2 MATERIALS AND METHODS

### 2.1 Mice

The mice with a targeted inactivation of AGKO on C57BL/6 background were obtained from Dr. Anthony d'Apice via Dr. Megan Sykes, Columbia University Medical Center, New York.^15^ AGKO mice were bred and maintained in microisolator cages on racks with HEPA‐filtered air blown into each cage and all animal protocols were approved by the University of North Carolina Institutional Animal Care and Use Committee (IACUC). Euthanasia was performed by anesthetizing animals with an intraperitoneal injection of 1.25% avertin (125–250 mg/kg body weight) followed by cervical dislocation.

### 2.2 Sensitization to alpha‐gal and food challenge

Eight‐ to 10‐week old AGKO mice were injected intradermally with 50 μg of *Amlyomma americanum* TSGE or saline on Days 0, 7, 21, 28, 42, and 49 (see Supplementary materials for details on preparation of TSGE). Mice were bled on Days 0, 7, 21, 28, and 56 to quantitate total and specific IgE. Mice sensitized to alpha‐gal and control mice were challenged on Day 60–64 (11–15 days following final tick sensitization at Day 49) with 400 mg of cooked pork kidney (Mutschler's Hausmacher specialization, Germany) homogenate in phosphate buffered saline (PBS). Second and third food challenges were performed 4 and 8 days later on mice that did not meet the 2°C temperature drop upon initial challenge. Body temperature was measured with a rectal probe (Braintree Scientific Inc) before the challenge and every 15 min up to 2 h after the challenge. Mice were conditioned to rectal probe insertion before the food challenge to mitigate temperature variation induced by insertion of the rectal probe. Allergy signs were scored on a 0 to 5‐point scale as follows: 0, no signs; 1, scratching around the nose and head; 2, reduced activity with pilar erecti or diarrhea; 3, labored breathing; 4, minimal responsiveness to prodding and 5, death. Animals showing minimal responsiveness to prodding were euthanized to relieve pain and not allowed to proceed to condition 5 if possible. Further, if a temperature difference of more than 2°C following the food challenge was observed, mice were sacrificed to collect blood and the spleen. Splenocytes from three mice were included on initial challenge and two mice from each of subsequent food challenges. Enzyme‐linked immunosorbent assay (ELISA) was performed to quantitate mouse mast cell protease (MMCP‐1) (eBioscience) according to the manufacturer's instructions.

### 2.3 Quantitation of total and specific immunoglobulins

Nunc Maxisorp plates were coated with capture antibody (rat antimouse IgE, 2 μg/ml, SouthernBiotech) or the antigen of interest, such as cetuximab (20 μg/ml) and TSGE (20 μg/ml) in carbonate‐bicarbonate coating buffer to quantitate total IgE, alpha‐gal sIgE, and TSGE sIgE, respectively. Plates received four washes with PBS containing 0.05% Tween 20 (PBST) and were blocked with 3% FBS in PBST. ELISAs were detected with horseradish peroxidase (HRP)‐conjugated goat‐antimouse IgE‐HRP, 3,3',5,5'‐Tetramethylbenzidine Peroxidase Substrate and Stop Solution (KPL) was used to develop an enzymatic colored reaction. Plates were read on an Epoch Microplate Spectrophotometer (BioTek Instruments) and analyzed using Gen5 software.

### 2.4 Statistical analysis

Data were analyzed using GraphPad Prism 7 (La Jolla CA). The Mann‐Whitney test was performed for single comparison. For grouped analysis, multiple *T* test was performed along with Holm–Sidak multiple comparison test for pairwise comparison. Fisher's exact test was used to calculated relative risk and statistical significance. The *p* value less than 0.05 was considered statistically significant.

## 3 RESULTS AND DISCUSSION

### 3.1 Alpha‐gal sensitization with TSGE in AGKO mice

**Figure 1:**
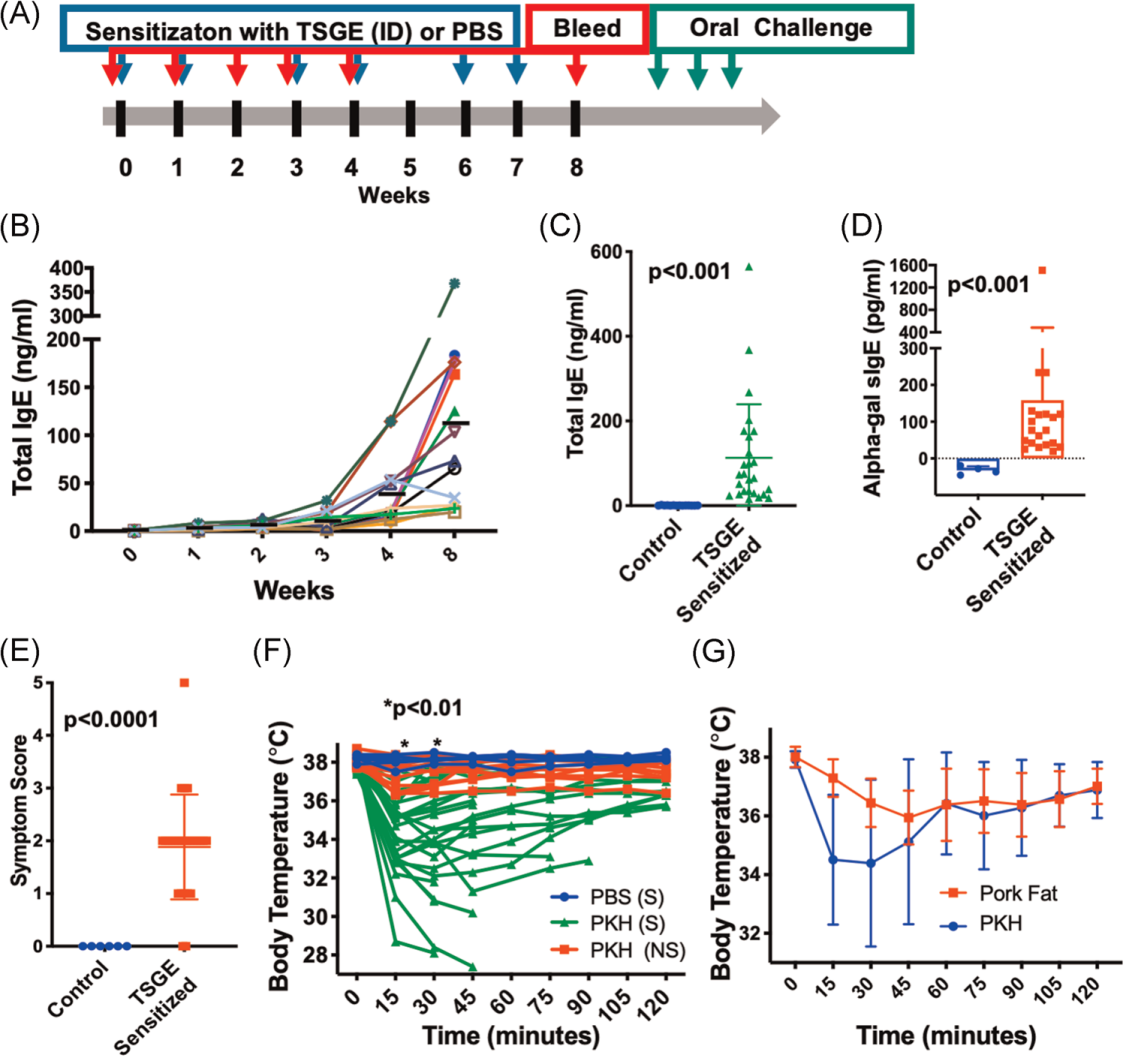
Alpha‐gal sensitization and red meat challenge in AGKO mice. (A) Schematic of alpha‐gal sensitization with intradermal injection of Amblyomma americanum tick salivary gland extract (TSGE) and oral challenge. (B) Total IgE kinetics in individual mice following TSGE injection in a representative experiment; black dash indicates mean (*N* = 12). (C) Quantitation of total IgE in control (N = 14) and TSGE‐sensitized mice (*N* = 26). (D) Quantitation of alpha‐gal specific IgE in control (*N* = 4) and TSGE‐sensitized mice (*N* = 20). The scatter plots (C‐D) show mean with 95% confidence interval on Day 56. (E–F) Allergic response post‐oral challenge with 400 mg of cooked pork kidney homogenates (PKH) or phosphate‐buffered saline (PBS) in individual mice (controls: *N* = 6; TSGE‐sensitized: *N* = 26). Allergic symptoms were scored at 30 min post‐challenge and show mean with standard deviation. Body temperature was recorded at baseline and post‐oral challenge with either PBS or PKH in sensitized (S) and control (NS) mice. A drop in body temperature was significant at 15 and 30 min in control versus TSGE‐sensitized mice when challenged with PKH. (G) Challenge of TSGE‐sensitized mice with pork fat (*N* = 11) delays allergic responses in comparison to PKH (*N* = 26). Mice were included in the analyses for Figure 1E–G at the food challenge when a >2°C temperature drop occurred. The Mann–Whitney test was performed for single comparison. For grouped analysis, multiple *T* test along with Holm–Sidak multiple comparison test was performed for pairwise comparison. AGKO, inactivation of the alpha‐1,3‐galactosyltransferase gene; IgE, immunoglobulin E

To mimic the absence of alpha‐gal in humans, we treated 8–10 week old AGKO mice intradermally (id) with 50 μg of partially blood fed TSGE or PBS. An intradermal injection was used to replicate a tick bite. Mice were bled on Days 0, 7, 21, 28, and 56 to quantitate total IgE (Figure 1A). We observed a gradual increase in total IgE level following each TSGE injection (Figure 1B). By Week 8, total IgE in TSGE‐injected mice was 190‐fold higher than in control animals (113.2 ± 24.77 vs. 0.60 ± 0.12 ng/ml; *p* < 0.001, Figure 1C). By comparison, direct *Aa* tick attachment to C3H/HeN mice led to a more robust total IgE response at two‐weeks post‐tick feeding (7318 ± 2905 ng/ml) but the hazards of maintaining live ticks in the animal care facility favored use of injected TSGE (Figure S1). Consistent with reports of *Aa* bites in humans leading to AGS, alpha‐gal sIgE (158.4 ± 72.43 pg/ml) was detected in TSGE‐injected mice at Day 56 (Figure 1D). Chandrasekhar and colleagues reported the presence of alpha‐gal sIgE in AGKO mice following immunization, however, they augmented *Aa* whole tick extract with chemically synthesized alpha‐gal containing BSA to generate alpha‐gal sIgE.^16^ While their approach uses whole tick extract as an adjuvant, we have not found this necessary. Our protocol utilizes three additional inoculations with antigen for a total of six inoculations.

We report here that *ex vivo* inoculation of splenocytes from control mice with TSGE caused an increase in the expression of CD69, a surrogate marker of early cell activation on B220^+^CD19^+^ B cells (Figure S2). However, treatment of splenocytes with TSGE from TSGE‐sensitized AGKO mice led to greater activation of B220^+^CD19^+^ B cells, providing evidence of a recall response following antigen exposure. These findings, together with the report of alpha‐gal containing epitopes in ticks linked to AGS, suggest that the presence of alpha‐gal in tick saliva might be required for sensitization.^10^, ^12^, ^14^ Alpha‐gal in the salivary compartment may not necessarily have to derive from a blood meal and could be endogenous, present in the microbiome, or induced during feeding.^10^

### 3.2 TSGE‐sensitized AGKO mice show a hypersensitive reaction following oral exposure to red meat

In contrast to most food allergies where the culprit antigens are a protein epitope that causes an immediate hypersensitivity reaction, in AGS the allergic reaction is directed against a carbohydrate moiety, and for reasons that are still not clear, typically delayed 2–6 h after the ingestion of meat.^3^, ^4^ Consumption of pork kidney, however, causes a shorter delay before symptoms (<2 h) and more consistent reactions—likely owing to the high alpha‐gal content in heavily glycosylated proteins, angiotensin I‐converting enzyme (ACE I) and aminopeptidase N (AP‐N) present in pork kidney.^4^, ^17^, ^18^ Therefore, we orally challenged TSGE‐sensitized AGKO mice with 400 mg of cooked pork kidney homogenate (PKH) (Mutschler's Hausmacher specialization) in PBS. Allergic signs were scored on a 0‐ to 5‐point scale (see Section Methods, 2). Following PKH challenge, all but one mouse showed mild allergic signs, such as itching and swelling in areas of the nose and mouth. Seventy‐three percent of TSGE sensitized mice showed moderate allergic signs, such as reduced activity and labored breathing (Figure 1E). We did not detect severe allergic signs, such as minimal responsiveness to prodding except for one mouse and the animal was culled to relieve the pain as required by the IACUC.

We further measured core body temperature with a rectal thermometer before the challenge and every 15 min for up to 2 h after the challenge as an objective measure of a systemic allergic reaction. A drop in core body temperature of ≥2°C was considered to indicate anaphylaxis. We observed a drop in body temperature of 2°C or more in 55.5% of mice following first exposure of PKH. When the drop in body temperature was <2°C, mice were re‐challenged with PKH after a one week rest interval. Greater than 50% of re‐challenged mice had decreases in core body temperature of ≥2°C following second exposure to PKH. Figure 1F represents the data set of individual mice showing the peak decline in body temperature in one of those two exposures. Body temperature reached its nadir at 30 min after the challenge and was significantly different than TSGE‐sensitized mice challenged with PBS or non‐sensitized mice challenged with PKH (*p* < .01 for both; Figure 1F). We observed a more immediate onset of reaction in alpha‐gal allergic mice following challenge with PKH, which is consistent with AGS in humans following consumption of mammalian innards and organ meats.^3^, ^4^ Equally, variability in the appearance and magnitude of allergic signs noted in our mouse model parallels the variability of the magnitude and timing of the allergic response to alpha‐gal reported in human subjects with AGS.^19^

Serum levels of MMCP‐1 were measured by ELISA (eBioscience) 30 min after peak drop in body temperature. We observed an average of 4.3‐fold increase in MMCP‐1 levels in TSGE‐sensitized mice (6453 ± 8946 pg/ml) compared to control mice (mean 1486 ± 831.6 pg/ml) following PKH challenge (*p* = 0.6303). A nonsignificant increase in the level of MMCP‐1 may reflect mast cell heterogeneity, less of a role for mast cells in this model or a range of reaction severity.

Instead of the more immediate onset reactions following PKH challenge, TSGE‐sensitized AGKO mice challenged with pork fat (400 mg) exhibited core body temperature nadir 45 min post oral administration (Figure 1G). This is 1.5‐times longer than after PKH challenge and 3‐times longer than the timing of core body temperature drops following the administration of a conventional protein antigen, such as peanut.^20^ The decline in body temperature in the pork fat fed group was significantly different than the PKH fed group at 15‐ and 30‐min post‐challenge (*p* < 0 .01). Entrance of glycolipid into the peripheral circulation following fat digestion and absorption takes greater than 3 h and may be the primary reason for delayed onset of allergic reactions in AGS.^21^

### 3.3 Gender influences the hypersensitive reaction to red meat in TSGE‐sensitized AGKO mice

**Figure 2:**
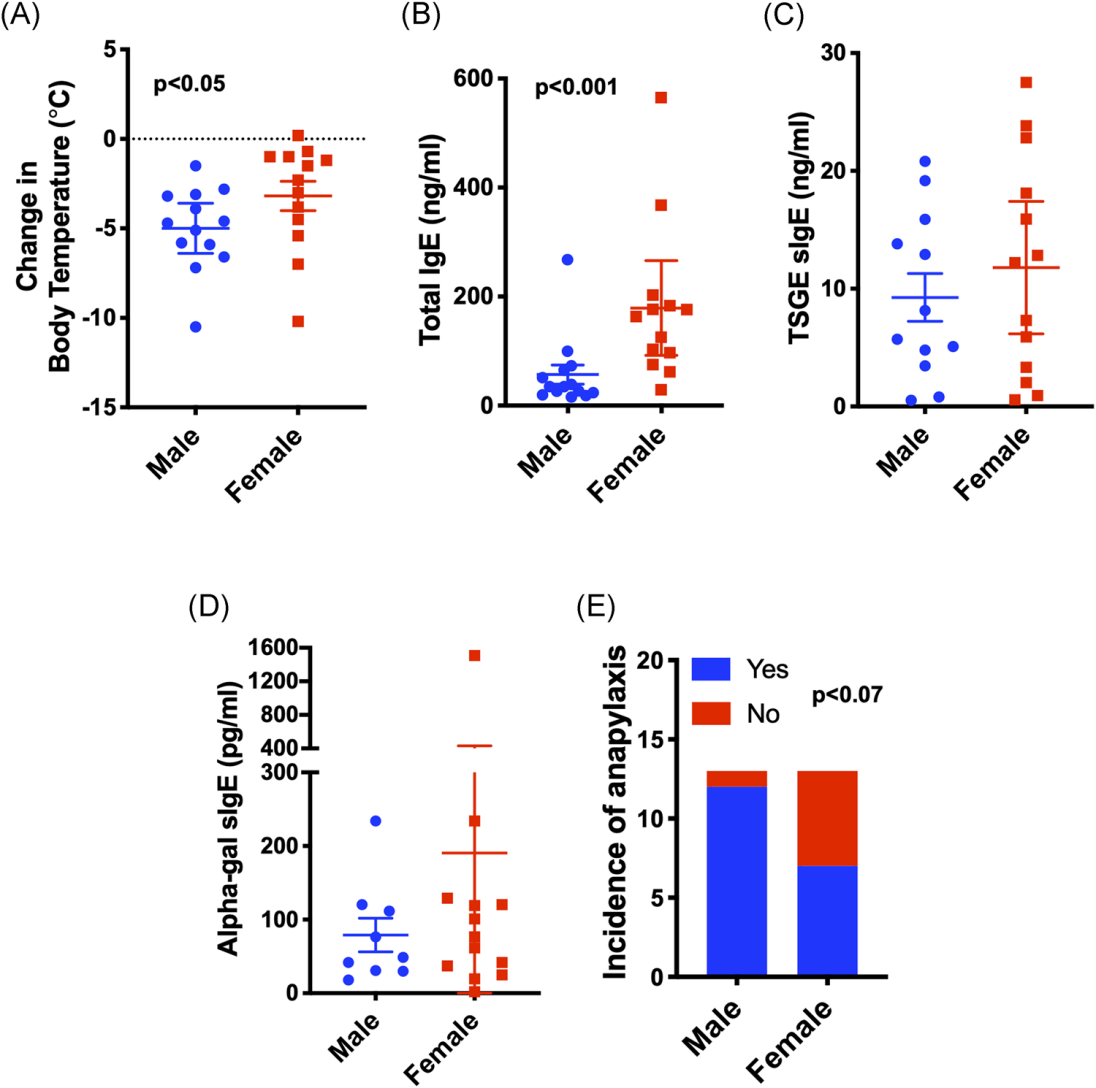
Sex difference in the frequency and severity of anaphylaxis in TSGE‐sensitized AKGO mice. (A) Change in body temperature following oral meat challenge (*N* = 13 for each sex). (B) Quantitation of total IgE (*N* = 13 for each sex). (C) Quantitation of TSGE specific IgE (male, *N* = 12; female, *N* = 13). (D) Quantitation of alpha‐gal specific IgE (male, *N* = 9; female *N* = 13). (E) Frequency of anaphylaxis following oral meat challenge (*N* = 13 for each sex). The scatter plots show mean with confidence interval. The Mann–Whitney test was performed for single comparison. Fisher's exact test was used to calculated relative risk and statistical significance. AGKO, inactivation of the alpha‐1,3‐galactosyltransferase gene; IgE, immunoglobulin E; TSGE, tick salivary gland extract

The titer of alpha‐gal sIgE does not predict reaction severity in humans with AGS; rather dose, fat content, and presence of co‐factors (alcohol, activity) affect the resulting clinical manifestations.^3^, ^4^ Of note, sensitized mice challenged with pork fat had a delay in the appearance of reaction signs in comparison with PKH‐challenged mice (Figure 1G). Moreover, in keeping with reactions to red meat in humans, the titer of alpha‐gal sIgE alone was not predictive of reaction severity in our murine model (Figure S4). Interestingly, male mice on average had a greater change in core body temperature following PKH challenge than females (−4.99 ± 0.64°C vs. −3.18 ± 0.82°C; *p* < 0.05; Figure 2A) despite having a significantly lower total IgE (56.98 ± 17.45 vs. 179.10 ± 39.86 ng/ml; *p* < 0.001; Figure 2B) and a similar level of tick sIgE (9.25 ± 2.03 vs. 11.79±2.58 ng/ml; *p* < 0.57; Figure 2C) and alpha‐gal sIgE (79.13 ± 22.84 vs. 190.50 ± 111.20 pg/ml; Figure 2D). Male mice were 1.7‐times more likely to have anaphylaxis than female mice (*p* < 0.07; Figure 2E). This murine model hints at potentially important sex‐related differences in the manifestation and severity of allergic responses in AGS. Differences in clinical signs and symptoms of AGS between the sexes have not been definitively established, although there is a report of increased incidence of sIgE to alpha‐gal in male patients.^22^

## 4 CONCLUSION

We demonstrate that intradermal injection of TSGE in AGKO mice induces alpha‐gal sIgE production, establishing a central role of tick bites in the development of AGS. Moreover, there was variability in the severity of the allergic response induced in TSGE‐sensitized, PKH‐challenged animals. Similarly, alpha‐gal allergic subjects do not always exhibit systemic reactions after red meat ingestion and the timing for the onset of symptoms appears to depend on the amount of meat consumed and its fat content.^3^, ^4^ A prior model using AGKO mice augmented *Aa* whole tick extract with chemically synthesized alpha‐gal containing bovine serum albumin to generate alpha‐gal sIgE.^16^ We demonstrate sensitization with TSGE alone and have previously reported the presence of alpha‐gal epitopes in the saliva of *Aa* fed on the blood of humans (naturally alpha‐gal deficient).^10^ Our results are consistent with the findings of Araujo et al. where saliva of *Amblyomma sculptum* containing alpha‐gal induced anti‐alpha‐gal antibody response; however, an allergic reaction to alpha‐gal containing food was not tested in their model.^14^ Uniquely, we show that oral challenge in sensitized mice with pork fat results in characteristic delayed allergic responses while ingestion of pork kidney, which contains high glycoprotein, causes a rapid reaction of increased severity reflective of AGS in humans.^4^ Importantly, these experiments do not establish that alpha‐gal is the allergen responsible for reactions on PKH. Figure S3 suggests that de‐glycosylation of alpha‐gal‐containing cetuximab decreases biologic activity in the mouse model. Future studies are being conducted to address this point through use of PKH from alpha‐gal deficient pigs. Further, titer of alpha‐gal sIgE does not predict the severity of the allergic reaction and this novel model reveals potentially important sex‐related differences as a co‐factor. Overall our mouse model recapitulates several aspects of AGS seen in humans and provides a unique platform to study the mechanism of mammalian meat allergy, the role of tick saliva in the development of alpha‐gal‐directed IgE and to explore immunotherapy‐based treatments.

## CONFLICT OF INTERESTS

The authors declare that there are no conflict of interests.

## AUTHOR CONTRIBUTIONS

Scott P. Commins, Shailesh K. Choudhary, and Shahid Karim designed the experiments. Shailesh K. Choudhary, Shivangi Choudhary, Gary Crispell, Surendra Raj Sharma, Claire T. Addison, Mike Kulis, Brian H. Herrin, and Susan E. Little performed the experiments. Shailesh K. Choudhary, Shahid Karim, Onyinye I. Iweala, Mike Kulis, Brian H. Herrin, Susan E. Little, and Scott P. Commins analyzed the experiments. Shailesh K. Choudhary, Onyinye I. Iweala, and Scott P. Commins wrote the manuscript.

## Supporting information

Supporting information.

## Data Availability

The data that support the findings of this study are available from the corresponding authors upon reasonable request.
