# Supplementary material for: Tick salivary gland extract induces alpha‐gal syndrome in alpha‐gal deficient mice
Source: Immun Inflamm Dis. 2021 May 25;9(3):984–90. doi: 10.1002/iid3.457 (PMC8342229; doi:10.1002/iid3.457)
Supplement: Supplementary file 1 — Supporting information. [file IID3-9-984-s001.docx]

**SUPPLEMENTARY MATERIALS, METHODS & DATA for**

**Tick salivary gland extract induces alpha-gal syndrome in alpha-gal deficient mice**

**Running title: Alpha-gal syndrome in alpha-gal deficient mice**

**Shailesh K. Choudhary^1^, Shahid Karim^2*^, Onyinye I. Iweala^1,3*^, Shivangi Choudhary^1^, Gary Crispell^2^, Surendra Raj Sharma^2^, Claire T. Addison^1^, Michael Kulis^3^, Brian H. Herrin^4^, Susan E. Little^5^, and Scott P. Commins^1,3^**

^1^Thurston Research Center, Division of Allergy, Immunology and Rheumatology, Department of Medicine, University of North Carolina, Chapel Hill, NC

^2^Center for Molecular and Cellular Biosciences, School of Biological, Environmental, and Earth Sciences, The University of Southern Mississippi, Hattiesburg, MS

^3^UNC Food Allergy Initiative, Department of Pediatrics, University of North Carolina, Chapel Hill, NC

^4^Department of Diagnostic Medicine and Pathobiology, College of Veterinary Medicine, Kansas State University, Manhattan, KS

^5^Department of Veterinary Pathobiology, Center for Veterinary Health Sciences, Oklahoma State University, Stillwater, OK

***authors contributed equally**

**Correspondence**

Scott P. Commins

University of North Carolina at Chapel Hill, CB#7280

Chapel Hill, NC 27599-7280

Email: [scommins@email.unc.edu](mailto:scommins@email.unc.edu)

Shailesh K. Choudhary

University of North Carolina at Chapel Hill, CB#7280

Chapel Hill, NC 27599-7280

Email: [schoudha@med.unc.edu](mailto:schoudha@med.unc.edu)

**Supplementary Materials and Methods**

Tick salivary gland extract (TSGE). TSGE was prepared by Dr. Karim and colleagues. Briefly, adult female ticks were blood-fed and dissected within 2 h of removal from host sheep. Tick tissues were dissected and washed in M-199 buffer. Tissues were stored at −80°C in 0.15 M Tris-HCl, pH 8.0, containing 0.3 M NaCl, 10% glycerol, and 1% protease inhibitor cocktail. Salivary glands were solubilized in a protein extraction buffer consisting of 0.5 M Tris-HCl, pH 8.0, 0.3 M NaCl, and 10% glycerol, and were then treated with 1% HALT protease inhibitor cocktail. The tissues were crushed using pestles and sonicated using a Bioruptor Pico (Diagenode, Denville, NJ, USA) sonication device for 10 full cycles of 30 s pulse/30 s rest at 4°C. Homogenates were centrifuged at 5,000 x g for 10 min at 4°C and the supernatants were collected. Protein concentrations were estimated using the Bradford method, and protein was stored at −80°C.

**Supplementary Figures**

**
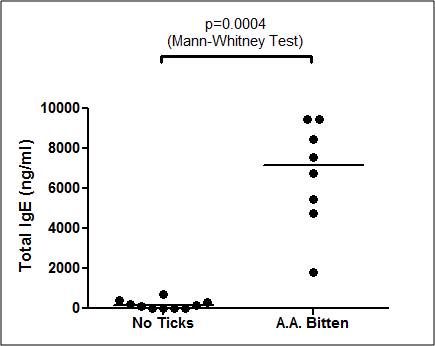
Figure S1.** *Amblyomma americanum* feeding on mice increases murine total IgE levels. Ticks were fed to repletion on C3H/HeN mice over 7-14 days using a small feeding chamber assembled on the mouse’s back. The data shown are compiled from two separate experiments. Circles represent individual mouse, lines are the median (p<0.001). Levels of total IgE appeared to correlate with mice that had more attached ticks in the chamber.

**Figure S2.** Salivary components of *Amblyomma americanum* activates B cells. Splenocytes from control or TSGE-sensitized AGKO mouse were treated with either saline or TSGE (10 μg/ml) and cultured for 24 hours *ex vivo* in RPMI 1640 medium with glutamine and HEPES containing 10% FBS, 1 mM sodium pyruvate, 1 mM MEM non-essential amino acid, 55 μM 2-mercaptoethanol and 1X penicillin-streptomycin. Splenocytes were stained with mouse B220 PE-Cy7, CD19-PerCP and CD69-APC and analyzed using a CyAn ADP flow cytometer and FlowJo 10 software (FlowJo LLC., Ashland, OR).


**Figure S3.** Scatter plot of change in temperature at time of PKH challenge in individual mice sensitized with TSGE plotted against alpha-gal sIgE. Data indicate a lack of association between reaction severity and alpha-gal sIgE (Spearman Test r = -0.1425; P = 0.5378).
